# Supplementary figures and images for: Microbial community composition across a coastal hydrological system affected by submarine groundwater discharge (SGD)
Source: PLoS One. 2020 Jun 29;15(6):e0235235. doi: 10.1371/journal.pone.0235235 (PMC7323985; doi:10.1371/journal.pone.0235235)

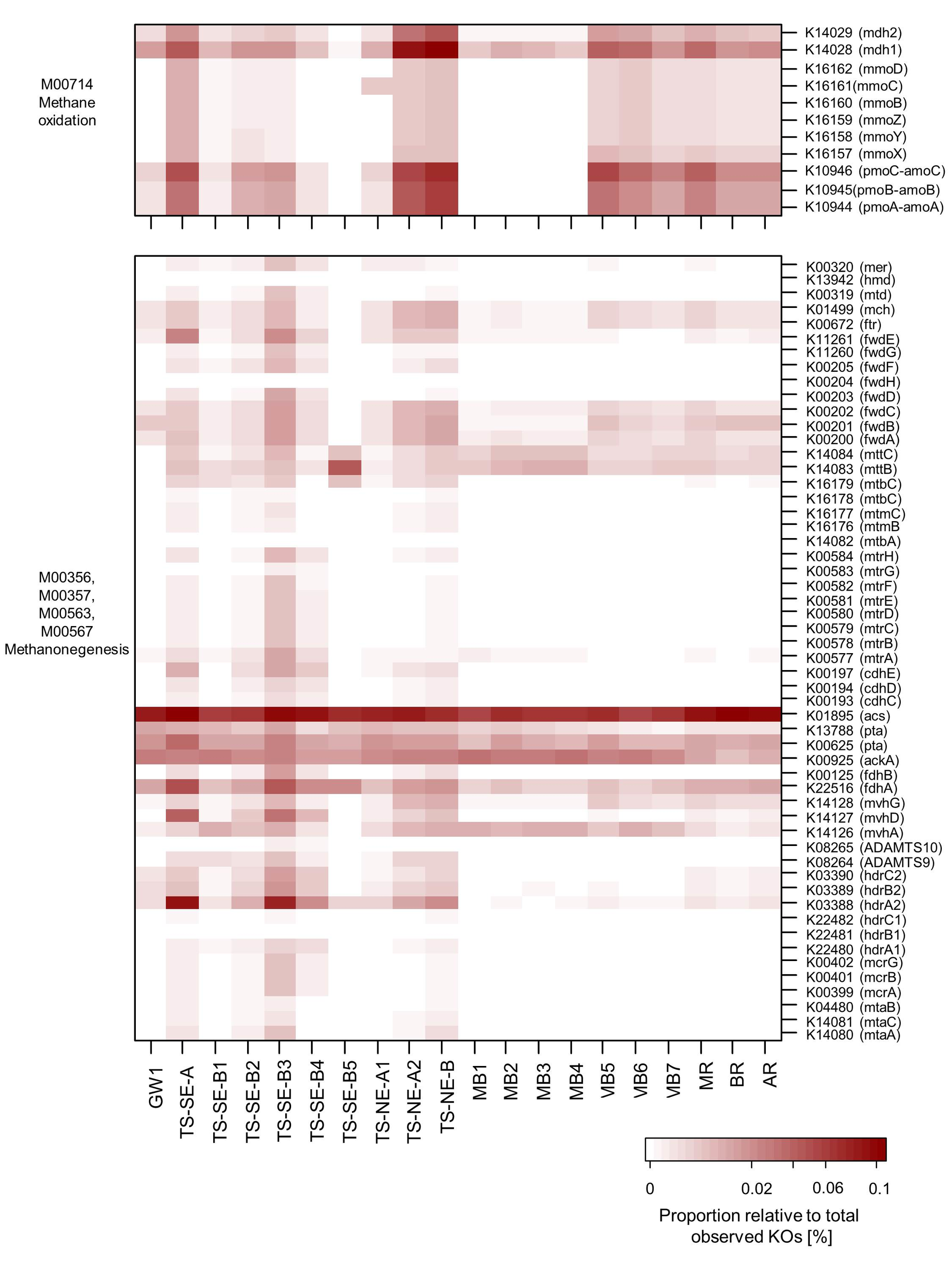

Supplement: S1 Fig — M00356: methanol to methane; M00357: acetate to methane; M00563: methylamine/dimethylamine/trimethylamine to methane; M00567: CO2 to methane. (TIF) [file pone.0235235.s001.tif]
