# Supplementary material for: Microbial community composition across a coastal hydrological system affected by submarine groundwater discharge (SGD)
Source: PLoS One. 2020 Jun 29;15(6):e0235235. doi: 10.1371/journal.pone.0235235 (PMC7323985; doi:10.1371/journal.pone.0235235)
Supplement: S1 Table — (DOCX) [file pone.0235235.s002.docx]

|  | Surface samples | | | Subsurface samples | | |
| --- | --- | --- | --- | --- | --- | --- |
|  | nOTU | Shannon | invS | nOTU | Shannon | invS |
| Salinity | -0.854 | -0.560 | -0.402 | 0.195 | 0.391 | 0.425 |
| DO | -0.536 | 0.050 | 0.042 | 0.120 | 0.313 | 0.289 |
| Temperature | -0.218 | -0.142 | -0.218 | 0.512 | 0.171 | 0.171 |
| NO_3_^-^ | 0.800 | 0.667 | 0.550 | -0.024 | 0.167 | 0.167 |
| NH_4_^+^ | 0.717 | 0.500 | 0.383 | 0.381 | 0.048 | 0.048 |
| PO_4_^3-^ | 0.617 | 0.250 | 0.283 | 0.500 | 0.524 | 0.524 |
| DON | 0.100 | 0.117 | 0.383 | -0.309 | -0.619 | -0.619 |

**S1 Table. Spearman correlation between alpha diversity and observed environmental parameters**
