# Supplementary material for: Microbial community composition across a coastal hydrological system affected by submarine groundwater discharge (SGD)
Source: PLoS One. 2020 Jun 29;15(6):e0235235. doi: 10.1371/journal.pone.0235235 (PMC7323985; doi:10.1371/journal.pone.0235235)
Supplement: Attachment — Submitted filename: RevisionPLOS ONE.pdf [file pone.0235235.s003.pdf]

Revision of the manuscript "*Microbial community composition across a coastal hydrological system affected by submarine groundwater discharge (SGD)*" by Adyasari *et al.*

The paper of Adyasari *et al.* explores the spatial dynamics of prokaryotic communities along a coastal hydrological gradient in the Mobile Bay, USA, which is influenced by submarine groundwater discharge (SGD), as well as its links to environmental conditions. To do so, they collect water samples from 20 sites, including fresh groundwater, two vertical profiles of porewater in the intertidal zones, river water and bay water. The bacterial community composition is characterized by sequencing of the 16S rRNA gene, and the physicochemical parameters measured include salinity, temperature, pH, dissolved oxygen and concentration of inorganic nutrients. In addition, they estimate the functional diversity from the taxonomic data using the Tax4Fun2 method.

Their results show pronounced changes in the structure of the prokaryotic assemblages between the different habitats covered, and a high vertical heterogeneity within each piezometer. The environmental variables most strongly related to the observed variations in microbial communities are salinity and nitrate, and they find higher diversity in the intertidal pore waters than in the fresh groundwater and in surface river or seawater. The authors provide a detailed and complete description of the taxonomic groups detected. Based on the functional analysis, the authors suggest that subsurface microbial communities may play a large role in nitrogen cycling, thus affecting the nutrients delivered to the bay. The authors conclude that that their results support previous studies in the area showing that SGD supports productivity.

The research presented here is relevant because it provides information about the spatial variations in microbial communities in subterranean estuaries, largely undersampled for microbial studies in comparison to other surface aquatic habitats. The study is also of relevance since it compares the original groundwater, river water, bay water and pore waters located in a single area, which allows a comprehensive exploration of the microbial diversity in this groundwater-marine interface, which is also one of the least studied ecotones in terms of microbial ecology and biogeography. The study shows how complex and heterogeneous groundwater microbial assemblages are, even at such small spatial scales. Given that groundwater aquifers appear to hide a vast diversity of unknown taxa, any study providing insight on the subsurface microbial inhabitants is of interest. The manuscript is well written, the results are well presented and clearly explained, and the discussion is well contextualized.

In my opinion, however, the study needs to address some issues, and to acknowledge some of its limitations. For example, this type of intertidal systems may be extremely variable, not only depending on tidal fluctuations but also on hydrologic regimes. As this paper presents a single snap-shot of the system (one sampling day), the conclusions must be taken with caution and the existence of large temporal variations in the reported patterns at different scales must be acknowledged and discussed in the Discussion section. In my opinion, the manuscript should also better highlight the lack of studies targeting groundwater estuaries, and should put their results in the context of such previous knowledge; although to my knowledge very few studies have explored the microbiology in these type of systems, there are some that have not been included in the manuscript and thus should be cited (see below). Also, the authors find important proportions of Archaea, and to my knowledge not much is known about archaea in groundwaters. A better contextualization of these results in view of the existing literature would render the manuscript more interesting and novel, as there is truly a lack of studies focusing on these sites that appear to be important hotspots of biogeochemical activity.

Given the large spatial heterogeneity reported in the vertical pore water profiles, I think that it would be interesting to address the drivers of the studied communities including only the subsurface samples, as it is expectable that salinity will be one of the drivers explaining the majority of the variation when river and bay samples are included. However, based on the NMDS and the PCA, it does not seem that salinity is the main driver of the variations across/within the subsurface samples, is it? I suggest that the analysis is performed in parallel including all samples together but also considering only the subsurface ones.

Finally, as the sampling covers a nice groundwater-river-brackish gradient, I think it would be interesting to explore a little bit more whether the authors can find indicator taxa associated to a given habitat, or to which extent the different ground- and porewater samples are comprised of typical marine or typical freshwater taxa (see comments below).

Although I see that this manuscript provides interesting and detailed information of the studied assemblages, I recommend that these and other issues (see below) are addressed for this study to be published in PLOS ONE.

Major comments:

1. Overall, I see the relevance of the research and the interest of the sampling design, but in my opinion the manuscript would largely benefit from modifying partially the Introduction and the Discussion so that the relevance, novelty or need of conducting the present research is better contextualized and justified. The study of groundwater microbial ecology is gaining increasing attention, and there have been many recent

papers on groundwater communities, including some targeting subterranean estuaries (e.g. Héry et al. 2014 FEMS, Chen et al. 2019 STOTEN, Sang et al 2018 Sci Reports). A comparison to such previous studies would highlight the similarities or differences with such previous investigations, thus underlying the specific contributions of this investigation.

2. As mentioned before, the authors have interesting information that is however not shown in the figures. For example, could they make a figure analogous to Figure 3 and Figure 6 but considering only the subsurface communities? This would help better identify the environmental gradients that drive the pronounced spatial heterogeneity found within and across these communities. Including the river and bay water is interesting to place subsurface communities within the whole hydrologic gradient, but it is well known that salinity drives bacterial communities in the surface fresh-marine transitions... at the subsurface, this may be less clear and assemblages be strongly influenced by other gradients such as dissolved oxygen, which will be more clearly visualized when only those samples are considered.
3. Similarly, the fact that the surface seawater and freshwater are also included allows for a deeper exploration of e.g. typical groundwater indicators (*indval* analysis), or to explore the percentage of 'river' or 'marine' taxa that comprise the studied subsurface communities. There is also a recent study (Rocca et al 2020 Ecology) that is relevant in this regard.

Specific comments:

-One of the main conclusions stated in the abstract is not easily derived from the results reported in the abstract: 'Overall, our study supported previous observations in the area highlighting the role of SGD in Mobile Bay's productivity'. This does not seem to be the main conclusion of the paper as nothing has been shown regarding the productivity in the Bay. I suggest that the end of the abstract is rewritten to deliver a more specific message that can be directly derived from the presented results.

-It is not clear why so many different bay water samples were taken. What was the purpose of this? Are they supposed to cover sites of different SGD influence? Based on temperature, for example, it seems that they are quite different in terms of salinity... something should be said about this in the methods section (to justify the choice of bay sites).

-Line 110: Please indicate how was GW1 collected. Is this groundwater sample supposed to be indicative of the groundwater in the area? Please indicate so.

-Line 127: How where the duplicate samples treated for the different analyses? Where they averaged? Please explain it. The duplicates are not shown in the figures, and I think it would be interesting to visually see whether the individual replicates cluster together in the ordination graphs

-Line 221-223: I find very interesting the large variations in taxonomic richness and diversity found within the TS-SE vertical profile. Something should be said about this. Where these changes in taxonomic diversity explained by any of the measured environmental parameters?

-Line 272: The figures should be explained in the order of appearance, so the authors should either redo the figures or reorganize the text. In my opinion, the general composition at the class level should be explained together with the explanation of Figure 5, focusing on the main differences between river, bay water and groundwater assemblages. Then, the current subsection (line 272) could focus exclusively on the vertical dimension, adding, as suggested above, the composition in terms of order, or family, besides genera. Both things could be later combined in the Discussion, but the results section needs to follow the figure order. Otherwise it is confusing for the reader.

-Table 1. Please check the nutrient values for TS-SE4 and 5, and TS-NE1 and 2, as they are exactly the same.

-Figure 3. As suggested above, I think it would be interesting to look at these patterns considering only the subsurface samples

-Lines 237-246: This figure includes the taxonomic composition but nothing is said about it, only the Bray-Curtis dissimilarity is mentioned. Please describe the compositional patterns shown in the figure. The description of the figures should follow the sequence of appearance.

-Figure 7: if I understand it correctly, the grey area lumps together all the taxa that were not classified at the genus level. However, as this area is so big, wouldn't it be possible to assign it to the smaller taxonomic level possible in those cases? e.g. family.... Alternatively, I think it would be useful to draw the same profile in parallel to this one, grouping taxa at the e.g. order level, in order to differentiate it from Figure 5 but to show more visually the vertical variations in composition (which are not as clearly shown in Figure 5 because sites are clustered based on similarity and not along the vertical profile).

In other words, I suggest that you add a similar graph to the left of figure 7 that shows the whole taxonomic composition at a higher taxonomic rank (something in between the class level shown in Figure 5 and the genus level shown in Figure 7). This will help visualizing more

clearly the vertical changes in composition, and then you can go to the identified genera in more detail.

-Figure 7: Here and throughout the text, replace on genus/class level by 'at the genus/class level'

-Figure 8: The proportion is calculated relative to what? to the total identified KOs? Please specify in the legend.

-Line 272: Microbial community composition at the horizontal and vertical scales?

-Line 272: The figures should be explained in the order of appearance, so the authors should either redo the figures or reorganize the text. In my opinion, the general composition at the class level should be explained together with the explanation of Figure 5, focusing on the main differences between river, bay water and groundwater assemblages. Then, the current subsection (line 272) could focus exclusively on the vertical dimension, adding, as suggested above, the composition in terms of order, or family, besides genera. Both things could be later combined in the Discussion, but the results section needs to follow the figure order. Otherwise it is confusing for the reader.

DISCUSSION: As explained above, in my opinion the manuscript would also benefit from expanding the discussion in order to compare the reported data with what has been done in previous studies targeting comparable systems. Although (at least to my knowledge) there are very few investigations exploring groundwater aquifers in coastal sites, these should be acknowledged and the results should be compared with them, in order to highlight the differences or similarities in the reported patterns (e.g. Héry et al. 2014 FEMS, Chen et al. 2019 STOTEN, Sang et al 2018 Sci Reports).

- Line 354: Changes in microbial community structure along the freshwater-marine continuum?

-Lines 364-372: This statement could be nicely shown if exploring the contribution to each subsurface community of typical groundwater, river water or marine water taxa. I expect that this would nicely show what are the mixing zones, and which are those sites where totally different communities arise, that do not result from the passive mixing of marine and freshwater taxa. There is accumulating evidence that microbial communities are largely structured by hydrologic connectivity (e.g. Ruiz-González et al 2015 Ecol Lett shows that freshwater bacterial communities are dominated by rare taxa transported from soils, some of which appear to grow in water). Rocca et al (2020 Ecology) is also relevant here ('*Rare microbial taxa emerge when communities collide: freshwater and marine microbiome responses to experimental mixing*').

There are other papers showing that the resuscitation of rare taxa can impact ecosystem processes such as CO_2 and methane production (Aanderud *et al.* 2015 Front. Microbiol, Stegen *et al.* 2015 Nature Communications), so that the rare taxa that appear in the studied pore water sites may play large roles in coastal biogeochemistry. The manuscript would benefit from an expansion of the discussion on this topic, on the role of hydrologic transport of microbial diversity and the selective growth of some of the transported taxa when encountering new environmental conditions.

-Lines 432-440: To my knowledge, not much is known about Archaea in subsurface ecosystems, and in particular in subterranean estuaries. The authors should highlight what is known or not about the topic, in order to better highlight the specific contributions of the presented research.

-Line 444-445. Please rewrite this sentence to specify more clearly that NO3 is transformed to NH4 during transit through this site, which is then delivered to the Bay

-Line 446, replace 'and it is likely generated' with ',which are likely generated'

-Line 450: was related

-Line 458: depending on

REFERENCES CITED HERE

Aanderud, Z.T., Jones, S.E., Fierer, N. & Lennon, J.F. (2015) Resuscitation of the rare biosphere contributes to pulses of ecosystem activity. *Frontiers in Microbiology*, **6**, 24.

Héry, M., Volant, A., Garing, C., Luguot, L., Poulichet, F.E. & Gouze, P. (2014) Diversity and geochemical structuring of bacterial communities along a salinity gradient in a carbonate aquifer subject to seawater intrusion. *Fems Microbiology Ecology*, **90**, 922–934.

Rocca, J.D., Simonin, M., Bernhardt, E.S., Washburne, A.D. & Wright, J.J. (2020) Rare microbial taxa emerge when communities collide: freshwater and marine microbiome responses to experimental mixing. *Ecology*, **101**, e02956.

Ruiz-González, C., Niño-García, J.P. & del Giorgio, P.A. (2015) Terrestrial origin of bacterial communities in complex boreal freshwater networks. *Ecology Letters*, **18**, 1198-1206.

Sang, S., Zhang, X., Dai, H., Hu, B.X., Ou, H. & Sun, L. (2018) Diversity and predictive metabolic pathways of the prokaryotic microbial community along a groundwater salinity gradient of the Pearl River Delta, China *Scientific Reports*, **8**, 17317.

Stegen, J.C., Fredrickson, J.K., Wilkins, M.J., Konopka, A.E., Nelson, W.C., Arntzen, E.V., Chrisler, W.B., Chu, R.K., Danczak, R.E., Fansler, S.J., Kennedy, D.W., Resch, C.T. & Tfaily, M. (2016) Groundwater–surface water mixing shifts ecological assembly processes and stimulates organic carbon turnover. *Nature Communications*, **7**, 11237.
